# There is no human interactome

**DOI:** 10.1186/s13059-016-0913-4

**Published:** 2016-03-14

**Authors:** Michael P. Washburn

**Affiliations:** Stowers Institute for Medical Research, Kansas City, MO 64110 USA; Department of Pathology and Laboratory Medicine, The University of Kansas Medical Center, 3901 Rainbow Boulevard, Kansas City, KS 66160 USA

## Abstract

Protein complexes are dynamic. A new analysis of two quantitative proteomic datasets reveals cell type-specific changes in the stoichiometry of complexes, which often involve paralog switching.

Please see related Research article: www.dx.doi.org/10.1186/s13059-016-0912-5

## Introduction

The function of a protein is often tied to its interactions, and many proteins function as components of large multiprotein complexes. Multiprotein complexes also will connect to each other in a cell to carry out coordinated biological functions. Every cell has a network of protein interactions, where these connections within and between proteins and complexes yield insights into cellular states. Large-scale studies have been conducted to define human protein interaction networks through the analysis of thousands of affinity purifications in multiple cell types. Two recent studies by Huttlin and colleagues, and Hein et al., have reported human interactomes constructed using data from HEK293T cells [1], and HeLa cells [2], respectively. Both of these studies reported thousands of protein interactions, presenting one picture of protein interaction networks and topology in these different cell types.

There is certainly value in these types of analyses, in which, for example, new interactions of disease-related proteins can be found and characterized [1]. However, there is no single, fixed human interactome. Instead, it is likely that the number of interaction networks might number in the thousands, perhaps even an infinite number. Protein complexes and interaction networks are context specific—an example is the different forms of the Mediator protein complex that are differentiated and dependent on the specific bait protein used for affinity purification [3]. Additionally, protein complexes are dynamic, and differ across cell types and according to cellular stimuli. Furthermore, human therapeutics can alter specific protein interaction networks [4]. Specialized ribosomes, which vary in their subunit composition, are emerging as key regulators of embryonic development [5]. The mammalian SWI/SNF protein complex (also named BAF) has a dedicated subunit composition that is required for embryonic stem cell maintenance and pluripotency [6]. The above are just some examples of multiple studies that support the claim that there is no single human interactome.

Analyses of interaction networks are multidisciplinary efforts. In most of the papers mentioned above, computational biologists, biochemists, cell biologists and proteomics scientists were needed to perform the studies. These studies are often classified as ‘systems biology’, but this classification sometimes obscures the diverse range of skills needed to undertake these studies. For example, in 2013 a group at the European Molecular Biology Laboratory (EMBL) collaborated to analyze human nuclear pore complexes [7]. This integrated effort led to the discovery that the composition and stoichiometry of nuclear pore complexes varies across human cell lines. The collaborators proposed three different possible scenarios to explain their results: stoichiometric changes, subunit switching or competing interfaces [7]. The analyses of individual complexes such as SWI/SNF [6] and the nuclear pore complex [7] raise the question concerning how widespread might be the specific variance of protein complex composition.

## Computational analysis of stable and variable protein complexes

Several members of the same team that studied cell type-specific nuclear pores asked this question in a new study published in *Genome Biology* [8]. First, they built a protein complex resource from several database sources, including CORUM and COMPLEAT, that was then filtered to contain 279 protein complexes that each contains at least five distinct proteins, making a total of 2048 unique proteins. They then selected two large-scale quantitative proteomic datasets. One described an analysis of 11 human cell lines [9] and the other an analysis of mouse embryonic fibroblasts (MEFs) that had been induced into pluripotent stem cells (iPSCs) [10]. These original articles [9, 10] are both well executed, and detailed, quantitative proteomic studies, but it is important to bear in mind that they cover only a small fraction of the total number of possible cellular states. The authors then mapped the 279 protein complexes onto these two quantitative proteomic datasets and found that 182 were detected in one or the other of the datasets, and of these 116 were observed in both. A sizable portion of protein complex members were differentially expressed in both datasets, leading to the description of stable or variable protein complexes.

Over half of the 182 protein complexes analyzed were variable. More specifically, 102 of the complexes analyzed were variable, and 80 were stable. Stable complexes included the ribosome, the proteasome, mitochondrial protein complexes, and the exosome. However, some variability was seen in the ribosome, consistent with emerging evidence regarding the functional importance of specialized ribosomes [5]. By contrast, variable complexes included those involved in mRNA transport, vesicle-mediated transport and chromatin remodeling. Specific examples of variable complexes include TREX, COPII, COPI, SWI/SNF (BAF) and NuRD. From the quantitative proteomics datasets analyzed on different human cell lines [9], and iPSCs from MEFs [10], the major variable complexes were epigenetic regulators and transport systems.

These observations raise questions concerning how these variable complexes are regulated. Certainly, detailed and focused studies on each of the complexes are warranted in the future, but here the authors searched for general principles. They focused on the induced pluripotency dataset in mouse because gene expression data were available. Fewer than half of the cases of variant changes were likely attributable to transcriptional regulation, where protein and transcript abundance changed in the same direction at the same point in time. Almost two-thirds of the cases appear to be regulation at the level of translation or protein turnover. An analysis of structures from the Protein Data Bank suggested that stable interactions have structural properties different to those of variable interactions. Specifically, the authors suggest that variable interfaces are less hydrophobic than stable interfaces and might be more accessible to regulatory events such as phosphorylation.

## Paralog switching

Further analysis of the variable complexes revealed frequent paralog switching, where paralogs are genes produced through gene duplication in a genome and in these variable complexes one paralog would be replaced by another under certain circumstances. In the reprogramming dataset [10], the authors found 23 co-regulated paralog pairs, 16 of which had similar abundance differences—but in opposite directions. Two paralog switches found in the SWI/SNF (BAF) complex were the same paralog switches highlighted in a previous study showing the importance of specialized subunit composition for stem cell maintenance and pluripotency [6]. Additional reprogramming paralog switches occurred in the COPI, COPII and SNARE complexes, and COPII also had two paralog switches. The authors of the current study found a paralog switch in the NuRD chromatin-remodeling complex from the analysis of data from human cell lines [9]. A targeted proteomics analysis of MBD3-containing NuRD complexes from HEK293 cells verified their computational analysis.

Proteomics and genomic data existed for a limited number of the paralog switches. The authors analyzed these data to gain insight into the potential regulation of these switches. In most cases, changes in protein and transcript abundance correlated for one of the two paralogs, but, in the case of the SWI/SNF (BAF) complex, protein and transcript changes correlated for both paralogs. This suggests that there are probably several mechanisms for controlling such paralog switches.

## Concluding remarks

There is no single human interactome. There are many. How they are different and regulated is crucial for their understanding. Protein interaction networks are dynamic and context dependent. The differences in networks between cellular states are probably determined by key regulatory mechanisms for controlling these states. An excellent example reported in the recent *Genome Biology* study [8], and in previous work, is that of the SWI/SNF (BAF) complex and its importance in cellular reprogramming [6]. While the authors of the recent study [8] used two large-scale quantitative proteomic datasets, these studies represent a small fraction of the possible proteomes that could be analyzed. Development-, differentiation-, cell cycle-, normal- and disease- and drug-induced networks are all systems where variant complexes are likely to exist, and paralog switching might be a key regulatory mechanism. Clearly, how paralog switching itself is regulated will be an important area of future research. The final intriguing analysis carried out by the authors was a computational test to see whether the abundance of variable complex members can differentiate normal and cancer tissues. In the single situation presented this approach worked, but an analysis of a much larger scope, covering many more tissues, is warranted. However, it will be fascinating to see whether variable protein complex content is able to discriminate normal and diseased states.

## Abbreviations

iPSC: induced pluripotent stem cell
MEF: mouse embryonic fibroblast
